# Parental physical activity, safety perceptions and children’s independent mobility

**DOI:** 10.1186/1471-2458-13-584

**Published:** 2013-06-15

**Authors:** Maria Paula Santos, Andreia N Pizarro, Jorge Mota, Elisa A Marques

**Affiliations:** 1 Research Centre in Physical Activity, Health and Leisure (CIAFEL), Faculty of Sport, University of Porto, Rua Dr. Plácido Costa 91, Porto 4200-450, Portugal; 2 Higher Education Institute of Maia (ISMAI), Maia, Portugal

**Keywords:** Youth, Residence characteristics, Neighborhood, Environment, Physical activity, Outdoor activity

## Abstract

**Background:**

Parents are likely to be a basic influence on their children's behavior. There is an absence of information about the associations between parents' physical activity and perception of neighborhood environment with children’s independent mobility.

The purpose of this study is to examine the contribution of parental physical activity and perception of neighborhood safety to children’s independent mobility.

**Methods:**

In this cross-sectional study of 354 pupils and their parents, independent mobility, perceptions of neighborhood safety and physical activity were evaluated by questionnaire. Categorical principal components analyses were used to determine the underlying dimensions of both independent mobility and perceptions of neighborhood safety items.

**Results:**

The strongest predictor of independent mobility was the parental perception of sidewalk and street safety (ß = 0.132). Parent’s physical activity was also a significant predictor. The final model accounted for 13.0% of the variance.

**Conclusions:**

Parental perception of neighborhood safety and parents’ self reported physical activity might be associated with children’s independent mobility. Further research in this topic is needed to explore this possible association.

## Background

Time spent outdoors is positively associated with physical activity levels of children [1], and has been suggested as a proxy for physical activity [2]. However, opportunities for physical activity are being missed since children spend less time playing outdoors [3], and have lower participation rates in active transport [2,4]. Better understanding of the factors influencing children’s physical activity will support the development of successful interventions that stimulate an active lifestyle and diminish the time spent on inactive behaviors [5,6].

A range of factors have been postulated as potential influences on children’s physical activity and sedentary behaviors. Review studies show some evidence for associations between physical activity and demographic, psychosocial, behavioral, environmental and social factors among youth [5-7]. However, the role played by parents (parental modeling) and local neighborhood environments has been subject of an increasing body of research because such information would be useful to intervention development [8,9]. Although there is limited knowledge about factors related to independent mobility in children, research considered that lost of freedom to explore and achieve mastery over physical and social environment could limit children’s opportunities to develop healthy lifestyles, social networks and environmental competence and resilience [10,11]. While studies showed that children’s levels of independent mobility might influence their physical, social, cognitive and emotional development [12] and is significantly associated with physical activity [13] it has been suggested that compared with previous generations, children today are more restricted in their independent mobility [14] In particular, parents’ perception of harm from strangers and road safety are identified as major causes of parental anxiety [15], and such concerns may cause parents to restrict their children’s outdoor play and autonomous active transport [8,16]. Although parent physical activity behaviors (parent modeling) and neighborhood environments are likely to influence physical activity among youth [9,16-18], few studies have examined the associations with independent mobility. Studying this potential impact has critical implications for health promotion, as independent mobility is considered an important independent correlate of physical activity for both boys and girls [19], and may impact their physical, social, cognitive and emotional development [20]. Thus, environments that promote greater independent mobility in children may increase their physical activity levels and hence avoid missing out on the health benefits associated with regular physical activity during childhood and adolescence [21].

Therefore, this study aimed to examine the possible contribution of parental perception of neighborhood safety and parents’ physical activity level (parental modeling) to independent mobility among children.

## Methods

### Participants

This study is a secondary analyses of the baseline data from the SALTA Project (Environmental Support for Leisure and Active Transport), a longitudinal study in Porto area, Portugal, designed to examine environmental and social influences on PA in children and adolescents [22]. Two groups of participants were recruited: children (6^th^ grade students), and their parents.

All public middle-schools in Porto area (n = 65) were invited to take part in the study by letter, email and telephone. Fifty schools were excluded, 37 due to decline to participate and 13 did not reply to our invitation. From the 15 middle-schools that agreed to participate, 6 schools were not included due to logistical difficulties and this may have introduced selection bias. Thus, the final sample included 9 middle-schools, resulting in a total of 652 participants.

All participants were informed about the objectives of the study and parents or guardians of each participant provided written informed consent. Participants’ characteristics are listed in Table 1. This study was conducted according to the guidelines laid down in the Declaration of Helsinki. Ethical approval for this study was obtained from the Faculty of Sports, University of Porto, Scientific committee, the Portuguese Foundation for the Science and Technology and by the Regional section of the Ministry of Education.

**Table 1:** Participants characteristics

**Variable**	**Total participants (n = 354)**
Child age (years), mean (SD)	11.63 (0.85)
Child sex (male), n (%)	156 (44.1)
Child independent mobility, mean (SD)	2.11 (0.75)
Parent age, mean (SD)	40.19 (6.29)
Parent education	
Less than high school, n (%)	235 (66.4)
High school, n (%)	80 (22.6)
Some post-high school training or college, n (%)	7 (2)
Bachelor degree, n (%)	27 (7.6)
Higher education, n (%)	5 (1.4)
Parent PA	
Walking (min*number of days), mean (SD)	318.01 (310.94)
Moderate (min*number of days), mean (SD)	228.94 (325.42)
Vigorous-intensity (min*number of days), mean (SD)	224.81 (294.07)
Total MET-minutes/week^a^, mean (SD)	3763.00 (3403.73)
Parent perceptions of neighborhood safety	
Sidewalk and street safety^b^, mean (SD)	2.46 (0.55)
Fear of strangers, crime and traffic safety^c^, mean (SD)	1.92 (0.45)

^a^ Total MET-minutes/week = Walk (METs*min*days) + Moderate (METs*min*days) + Vigorous (METs*min*days); ^b^ statements included: Road safety is a concern to me, there is heavy traffic; I feel it is safe for my child goes out or play in the street during the day; I feel it would be safe for my child to go to a bus/train/metro stop during the night; I am concerned that my son can get robbed, when my child go out at night; I worry about strangers in my neighborhood; There is a high crime rate in my neighborhood; ^c^ statements included: There are crosswalks and pedestrian signals to help my kids cross streets safely; There are devices to slow down traffic (traffic lights or speed bumps); There are safe sidewalks for my kids travel from home to school; My neighborhood streets are well lit at night; There are many children playing or walking in my neighborhood.

Data collection took place during the 2010/2011 academic year.

### Measures

#### Children’s independent mobility

Independent mobility was assessed using the stem ‘How often are you allowed to go to the following places on your own or with friends (without an adult)’, which were part of a self-completed questionnaire. Eleven questions were included that were hypothesized to represent children's IM to visit a range of destinations in the neighborhood. These questions were based on common destinations reported in previous work [23] and on pilot data with 175 children (84 boys, 91 girls) from a large UK city, and has been previously used in other reports from the PEACH project [19,23].

As independent mobility was assessed using a 11-item, 5-point (Likert-type scale) response questionnaire, categorical (nonlinear) principal components analysis (CATPCA) was used to determine the underlying dimensions of the independent mobility items, as described below.

#### Family and demographic information and parental physical activity

Parent survey collected information regarding their relation to the child, age, education level, physical activity, and perceptions of neighborhood safety.

Previous week physical activity was self-reported using the short version of the International physical activity Questionnaire (IPAQ) [24]. The IPAQ has been evaluated in 14 studies and found to have good test-retest reliability and a modest Spearman correlation (r = 0.30) with PA measured by accelerometer [25] The IPAQ captures activity information on walking, moderate-intensity, and vigorous-intensity activities. The combined total physical activity score was obtained by the summation of the duration (in minutes) and frequency (days) for all levels of activities. According to the IPAQ scoring protocol, a measure of total volume of physical activity could be calculated by weighting each type of activity by its energy needs defined in METs (multiples of resting metabolic rate). Since there is still no established criteria, a minimum of at least 1500 MET-minutes/week of vigorous intensity physical activity or 3000 MET-minutes/week of a combination of walking, moderate and vigorous intensity might reflect a health enhancing physical activity level [24]. Physical activity was analyzed as a continuous outcome presented as MET-minutes/week, as described in detail elsewhere [24].

Questions about parental perceptions of neighborhood safety were adapted from the Neighborhood Environment Walkability Scale [26], and from previous studies [27,28]. These statements were related to perceptions about traffic density, road safety, strangers, sporting facilities and public transport in their local area (see Table 1). For each of the 11 items, parents could select from one of four options (Strongly Disagree to Strongly Agree). Items were recoded so that higher score indicates more positive perception of the environment. Data reduction was carried out using a CATPCA, as described below. This method is the nonlinear equivalent of standard PCA and reduces the observed variables to a number of uncorrelated principal components.

### Statistical analyses

Means and standard deviations (SD) for continuous variables and frequencies, and percentages for categorical variables were calculated to describe participants’ characteristics.

Multiple (five) imputations under the missing at random conditions were used to account for missing data in all variables that were included, according to procedures previously described [29,30].

To reduce both 11 items used to assess independent mobility and parental perceptions of neighborhood safety to a small number of composites with as little loss of information as possible, CATPCA was conducted using an ordinal analysis level as suggested when the number of categories is small [30].

An initial analysis was run to obtain a scree plot and eigenvalues for each component in the data. For independent mobility this process resulted in one component (Cronbach’s alpha = 0.904), which accounted for 51.11% of the variance. A second component/dimension was not retained as Cronbach’s alpha = −0.044 (eigenvalue = 0.962, % of variance = 8.74) suggested inadequate internal consistency for that particular factor. Regarding parental perception data, three components had eigenvalues over Kaiser’s criterion of 1 and in combination explained 56.26% of the variance. However, the third dimension was not retained as Cronbach’s alpha = 0.099 (eigenvalue = 1.098, % of variance = 9.98) suggested inadequate internal consistency for that particular factor. Thus, two factors were retained and object scores were calculated. The items that cluster on the same components suggested that component 1 represents sidewalk and street safety, and component 2 a fear of strangers, crime and traffic safety.

Multicollinearity between predictor variables was assessed by examining the variance of inflation factor and tolerance factor. Multivariate linear regression analysis was used to determine the influence of parental physical activity and neighborhood safety perception, expressed as continuous variables, on children’s independent mobility. Since differences in physical activity and independent mobility are well documented [19], all regression analyses were adjusted for age and sex.

Statistical analyses were carried out using SPSS 20.0 (SPSS Inc., Chicago, IL). The level of significance was set at *p* < 0.05.

## Results

Of the 652 eligible children and parents, survey was sent to all 652 parents, 354 (54%) of which were returned and included for analysis. In this analysis, children without their parent survey data were excluded (n = 298, 46%).

Table 1 shows the characteristics of the children and parents remaining in the analysis (n = 354). Of these students (aged 11.6 years), 44% were boys. Regarding independent mobility, the response 'I don't go there’ was not selected, therefore the variable was recorded such that a greater score represented greater independent mobility (mean scores ranged from 1 to 4). Children reported mean independent mobility scores of 2.11 (SD = 0.75). The majority of survey respondents were mothers (74%), while only 23.2% were children’s father. Twenty three percent of parents completed the high school, and had a mean total MET-minutes/week of 3,763.00, although total physical activity ranged from 0 to 15,878.40 MET-minutes/week.

Multiple linear regression results are shown in Table 2. After adjustment for individual characteristics (age and gender), parental total MET-minutes/week and the perception of sidewalk and street safety were significant predictors of children’s independent mobility, accounting for 13.0% of the variance (p < 0.001). The strongest predictor of independent mobility was the parental perception of sidewalk and street safety (ß = 0.132) while the dimension fear of strangers, crime and traffic safety was not a significant contributor.

**Table 2:** Independent predictors of IM from multiple linear regression analysis

**Predictor variable**	**ß**	***p***	**95% CI**
Total MET-minutes/week	0.104	0.041	1^-6^ – 6^-5^
Sidewalk and street safety	0.132	0.009	0.034 – 0.231
Fear of strangers, crime and traffic safety	0.061	0.225	−0.038 – 0.160

R^2^ = 0.13, overall p < 0.001; Model adjusted to child’s age and sex.

## Discussion

This study identified that parental perception of neighborhood safety and parents’ self-reported physical activity were associated with children’s independent mobility.

The positive impact of physically active role models has been documented in some studies exploring the influence of characteristics of the neighborhood on physical activity, but not in independent mobility [31]. According to present results, parents’ physical activity levels were positively associated with independent mobility in children. One possible explanation is the fact that more active parents may have better awareness of their neighborhood compared to those with low physical activity levels. In fact, parents’ decision about their children’s autonomy of movement does not depend merely on the environmental characteristics or on children’s ability to move autonomously. This decision habitually depends on their own personal concerns and subjective perception of the dangers that children may found without adult supervision [32]. Children’s independent mobility is defined as the opportunity for children to move freely in their environment without an accompanying adult, and is considered as an independent correlate of physical activity among children [19]. Independent mobility is measured in relation to spatial range or roaming range, and this measure can be determined by parents or caregivers in terms of the frontiers they set, or it can be the outcome of negotiations between children, parents or caregivers and even the community [10]. Autonomous exploration of the urban environment could provide children with opportunities for cognitive, social and physical development [33]. Adolescents who had been less autonomous during early childhood were more fearful about going out at night, felt lonelier and had weaker ties to their community [34]. Parents’ perceptions of their environment strongly shape their parenting practices [33]. For instance, parents often recognize that the neighborhood could promote opportunities for cognitive, social, and physical development of their children [11] and try to overcome their fears in order to enable and support independent mobility of their children.

Results also pointed out that parental perception of sidewalk and street safety (ß = 0.132) was the strongest predictor of independent mobility while the dimension fear of strangers, crime and traffic safety was not a significant contributor. Numerous studies have shown that restrictions on children’s independent mobility are mostly due to parental concern about road safety [15,35,36] and about strangers and social dangers [15,36,37]. Previous studies identified that parental perceptions of unsafe road environments were negatively associated with walking and cycling among 10–12-year-olds from Australia [38] and parental restriction of child’s active commuting from school [35]. However, evidence does not strongly support the relationship between neighborhood safety and children’s physical activity [39,40]. Parental restriction of their children’s independent mobility may be influenced by parental perceptions of local road safety, as well as the frequency of accidents within the neighborhood. Also Gielen et al. [41] found that parents restricted their children use of outdoor play settings because of ‘unsafe cars and trucks’, regardless of rate of child pedestrian injury there. But in many cases, the perception of road safety may be substantiated by accident statistics. Therefore road safety in the neighborhood could be a valid concern [15]. In fact, pedestrian unfriendly urban planning in many neighborhoods has reinforced the use of motorized vehicles, resulting in many parents and children being concerned about their safety due to fast-moving vehicles, irresponsible drivers and absence of crossing facilities or adequate pathways. These conditions, combined with the fact that many children and their parents are time challenged, support the choice of travel modes that are seen to be the least time consuming [13] and also limit children’s independent mobility.

The presence of sidewalks is often perceived as an environmental support for walking in adults [42]. Wilcox et al. [43], for example, found that the perceptions of neighborhood sidewalks were positively associated with physical activity. In addition, a Canadian study in a large sample of children found that the presence of good sidewalks/parks in the neighborhood was associated with less screen time and more physical activity [44]. Since parents are important facilitators of children’s physical activity and concerns about safety may restrict opportunities for active free-play and commuting, it is important to understand those parental concerns and other influences on children’s physical activity [2] and independent mobility. For example, a previous work found that perception of fewer social dangers and a more positive attitude towards child’s autonomy were the most influential variables on children’s independent mobility [33].

These findings might concur with the literature suggesting that a decline in children’s independent mobility increases the time that parents spend chauffeuring their children [13]. There is also evidence to support that parents’ activity behavior is important for children’s behavior, and the travel habits in childhood might foster norms that make the car as primary choice also in adult life [32].

There are limitations of this study that should be recognized, such as cross-sectional design, and the reliance on self-reported measures of independent mobility and parents’ physical activity levels. Missing data are a common problem in almost all data sets. Several studies have shown that simple methods such as complete-case analysis these led to loss in power and biased estimates [29,45]. Therefore, we used the multiple imputation method to account for missing data and minimize the non-response bias. Another important limitation is the lack of generalizability. In fact, despite the attempts to cover all schools within Porto area, only 7.2% of schools took part in the study. Nonetheless, results of present study contribute to improve the understanding on the link between parents’ individual and psychosocial variables to independent mobility.

To ensure children’s opportunity for independent mobility obvious solutions are to improve specific environmental attributes on residential areas, but those measures could be effective only if parents are encouraged to improve their physical activity levels and environmental quality perceptions. In fact, some evidence suggests that, when parents have a positive judgment regarding the potentiality of the environment, this could help to see their children’s independent mobility as a positive growth agent [11].

## Conclusion

In conclusion, results from the present study demonstrated that parental perception of neighborhood safety and parents’ self reported physical activity might be associated with children’s independent mobility. Further research in this topic is needed to explore this possible association.

## Competing interests

The authors have no conflict of interest to disclose.

## Authors’ contributions

MPS, AP and EM have made substantial contributions to study design, acquisition, analysis and interpretation of data; MPS and EM have been involved in drafting the manuscript. All authors revising it critically for important intellectual content and gave final approval of the version to be published.

## Pre-publication history

The pre-publication history for this paper can be accessed here:

http://www.biomedcentral.com/1471-2458/13/584/prepub
